# Ancillary ligand increases the efficiency of heteroleptic Ir-based triplet emitters in OLED devices

**DOI:** 10.1038/s41467-020-16091-1

**Published:** 2020-05-08

**Authors:** Seung-yeol Baek, Seung-Yeon Kwak, Seoung-Tae Kim, Kyu Young Hwang, Hyun Koo, Won-Joon Son, Byoungki Choi, Sunghan Kim, Hyeonho Choi, Mu-Hyun Baik

**Affiliations:** 1 Department of Chemistry, Korea Advanced Institute of Science and Technology (KAIST), Daejeon, 34141 Republic of Korea; 2 Center for Catalytic Hydrocarbon Functionalizations, Institute for Basic Science (IBS), Daejeon, 34141 Republic of Korea; 3 Samsung Advanced Institute of Technology (SAIT), Samsung Electronics Co., Ltd., Suwon, 16678 Republic of Korea; 4 Data and Information Technology (DIT) Center, Samsung Electronics, Hwaseong, 18448 Republic of Korea

**Keywords:** Optical materials, Single-molecule fluorescence

## Abstract

The excellent contrast ratio, visibility, and advantages in producing thin and light displays let organic light emitting diodes change the paradigm of the display industry. To improve future display technologies, higher electroluminescence efficiency is needed. Herein, the detailed study of the non-radiative decay mechanism employing density functional theory calculations is carried out and a simple, general strategy for the design of the ancillary ligand is formulated. It is shown that steric bulk properly directed towards the phenylisoquinoline ligands can significantly reduce the non-radiative decay rate.

## Abstract (web summary)

Though Ir-based dopants with ancillary ligands are attractive for realizing efficient organic light-emitting diodes, a strategy for designing these materials remains elusive. Here, the authors report a design strategy for heteroleptic triplet OLED emitters featuring functionalized ancillary ligands.

## Introduction

Organic light emitting diodes (OLEDs) revolutionized the display industry in the last two decades^1^. Despite being widely used, there is still much room for improvement in these devices, for example, in the energy efficiency of the current technology. One imperative market demand is for a deeper red with higher color purity, which proved challenging to satisfy with conventional emitters^2,3^. A new family of red dopants with longer wavelengths are needed, but simple energy gap law considerations explain that efficiency lowering due to increased non-radiative decay is inevitable^4,5^. Thus, luminescence efficiency is the most critical material property for commercial red dopants. As small changes in emission characteristics often exacerbate the efficiency drop, possible solutions considered contemplating host-dopant combinations for better orientation alignment of transition dipole moments^6,7^, or decorating the emitters with functional groups without too severely modifying the chromophore scaffolds^8^, but a decisive advance has not been achieved to date. A potential solution is to employ heteroleptic Ir-complexes carrying three bidentate ligands, of which two are mainly responsible for the luminescence, and one is a supporting ancillary ligand that is not directly involved in the phosphorescence. The goal is to eliminate the non-productive decay pathways by changing the ancillary ligand, thus exerting minimum impact on the luminescence properties^9^. Among various red dopants, Ir(III)-complexes carrying bidentate phenylpyridine (ppy) type ligands emerged as an important class of emitters^10^, and a typical ancillary ligand is an acetylacetone (acac) derivative^2^. Although the prospect of using the ancillary ligand to impose control over the chemical behavior of the dopant is attractive, successful implementations of rational design strategies involving the ancillary ligand are exceedingly rare^11^.

Herein employing a detailed computational model, we found that in addition to lengthening of the Ir–N bond, structural changes involving the angles of the coordination sphere contribute to the undesirable deactivation of the radiative state. Leveraging the insights from these precise computer models, we derived and experimentally confirmed a general design strategy. Whereas the DFT models are not necessarily accurate, they provide exact information that is easy to interpret and conceptualize.

## Results

### The non-radiative mechanism of heteroleptic Ir(III)-dopants

**Fig. 1:**
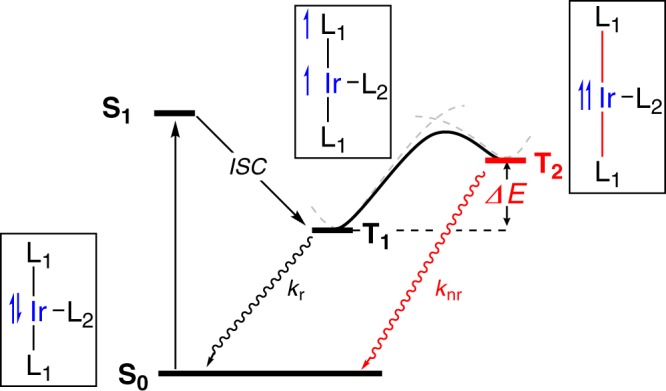
Qualitative energy diagram for the heteroleptic Ir(III)-dopants. The excitation of heteroleptic Ir-dopants (**S**_**0**_) affords to the singlet excited state (**S**_**1**_) which further undergoes the intersystem crossing to the triplet state (**T**_**1**_). This **T**_**1**_ state either emits light by phosphorescence (*k*_r_) or is excited to the **T**_**2**_ state that is responsible for the non-radiative decay (*k*_nr_).

It is well-established that the desired photophysical behavior relies on metal-to-ligand charge transfer (MLCT) excited states involving the π* orbitals of the ppy type ligand. Several pathways were discovered^12–14^, with the most critical non-radiative decay involving the **T**_**2**_ state, in which the metal-ligand bonds are slightly elongated, assisted by a structural motion of the phenylisoquinoline (piq) ligands. As illustrated in Fig. 1, this structural distortion leads to a triplet excited state in which unpaired electrons become localized at the metal center (^3^MC) that is responsible for non-radiative decay. Thus, a small energy gap (∆E) between the ^3^MLCT and ^3^MC states is expected to accelerate the non-radiative decay.

### Design strategy

**Fig. 2:**
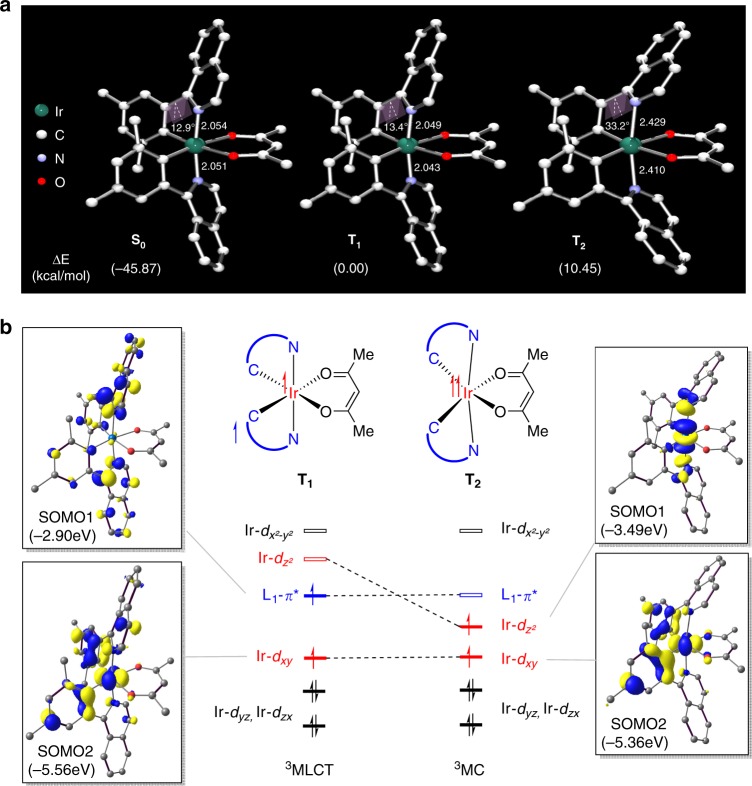
Various aspects in the excitation of the (piq)_2_Ir(acac). **a** DFT-optimized structures of the **S**_**0**_, **T**_**1**_ and **T**_**2**_ state of (piq)_2_Ir(acac). **b** Qualitative Walsh diagram illustrating the most important change in the ordering of the frontier orbitals in the two triplet states of Ir-complex. All bond lengths are in Å.

An ideal design strategy for effectively reducing the non-radiative decay rate of the heteroleptic Ir-dopants, which should improve the photoluminescence quantum yield while leaving the photophysical properties unaltered is to chemically modify the composition of the ancillary ligand. To identify the most promising implementation strategy, we employed density functional theory (DFT) calculations to locate accessible triplet excited state intermediates of the (piq)_2_Ir(acac) complex. Of particular interest is the triplet state corresponding to the ^3^MC state that is responsible for the non-radiative decay mechanism. For the prototype dopant complex, two different triplet states **T**_**1**_ and **T**_**2**_ were readily optimized as illustrated in Fig. 2a. The lowest energy triplet state (**T**_**1**_) displays a Ir-coordination geometry that is practically identical to that of the ground state (**S**_**0**_) with the two Ir–N bond lengths being 2.05 Å and the dihedral angle of phenylisoquinoline ligands being 13°. The second triplet excited state **T**_**2**_ is found to be 10.5 kcal mol^−1^ higher in energy than **T**_**1**_ and is characterized by distorted phenylisoquinoline ligands. The Ir–N bonds are notably elongated at 2.43 Å and the phenylisoquinoline ligands are notably distorted as illustrated in Fig. 2a, which of course affects the electronic structure tremendously. As depicted in Fig. 2b, **T**_**1**_ shows two singly occupied frontier orbitals **SOMO1** and **SOMO2** with significant contributions from the phenylisoquinoline ligands based π* and the metal-centered *d*_*xy*_ orbital, respectively. These frontier orbitals are expected for the metal-to-ligand-charge-transfer (^3^MLCT) state. The lengthening of the Ir–N bonds necessary to reach the **T**_**2**_ state stabilizes the high lying unoccupied antibonding orbital to an extent that the *d*_*z*²_-dominated LUMO of **T**_**1**_ becomes lower in energy than what was labeled as **SOMO1** in **T**_**1**_. As a result, the **SOMO1** of the **T**_**2**_ state displays a significant metal-centered (^3^MC) character. Being centered at the metal, the unpaired electron can take advantage of the strong spin-orbit coupling and reach the singlet state via spin-flip, establishing a non-radiative decay pathway.

**Fig. 3:**
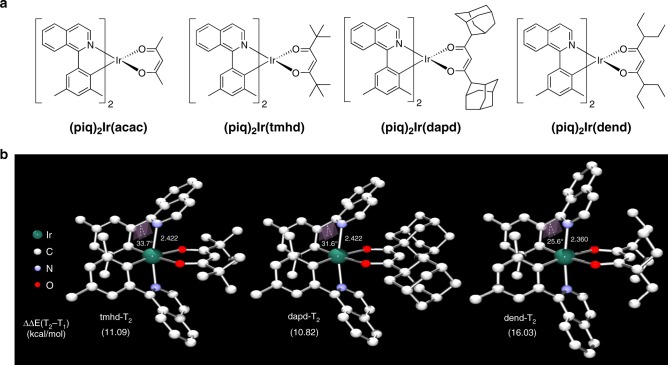
Rationally designed Ir(III)-dopants. **a** Chemical structures of Ir(III) complexes. **b** DFT-optimized ^3^MC state structures of (piq)_2_Ir(tmhd), (piq)_2_Ir(dapd), and (piq)_2_Ir(dend). All bond lengths are in Å.

These computational results suggest a simple strategy for inhibiting the non-radiative decay: The bending distortion of the isoquinoline fragment upon reaching the triplet state must be suppressed to improve the photoluminescence quantum yield. To achieve this goal without fundamentally changing the photophysics, we decided to modify the ancillary ligands by decorating them with sterically demanding functional groups that may block the distortion of phenylisoquinoline ligands. As depicted in Fig. 3a, four different ancillary ligands having different alkyl substituents were tested: (piq)_2_Ir(acac), (piq)_2_Ir(tmhd), (piq)_2_Ir(dapd), and (piq)_2_Ir(dend), where acac, tmhd, dapd, and dend represent acetylacetonate, 2,2,6,6-tetramethylheptane-3,5-dione, 1,3-di(adamantane-1-yl)propane-1,3-dione, and 3,7-diethylnonane-4,6-dione, respectively.

### DFT calculations on dopant candidates

The optimized structures of the **T**_**2**_ states of (piq)_2_Ir(tmhd), (piq)_2_Ir(dapd), and (piq)_2_Ir(dend) are illustrated in Fig. 3b. Compared to the parent complex (piq)_2_Ir(acac), where the isoquinoline distortion angle was 33.2° (Fig. 2a), the simple *tert*-butylation of the acac moiety in the tmhd ligand is not effective at a distortion angle of 33.7°. We were surprised to find that the adamantyl substituent in (piq)_2_Ir(dapd) afforded only a moderate change showing a distortion angle of 31.6°. A closer examination of the optimized structure shown in Fig. 3b revealed that while dapd is more sterically demanding than tmhd, the steric bulk is oriented in the acac-plane and away from the distortion axis of the isoquinoline moiety. Thus, the adamantyl groups become less effective than anticipated. The 3-pentyl substituents of the dend ligand have the highest impact on suppressing the bending motion of the isoquinoline fragment, and our calculations suggest the smallest distortion angle of 25.6°.

In addition to the degree of structural distortion in **T**_**2**_, the energy gap between the two triplet states **T**_**1**_ and **T**_**2**_ should have an impact on the non-radiative decay rate, as the efficiency of populating the **T**_**2**_ state should be inversely proportional to the energy gap. Our calculations suggest that (piq)_2_Ir(dend) has an energy gap of 16.0 kcal mol^−1^, which is 5.5 kcal mol^−1^ greater than the energy gap of 10.5 kcal mol^−1^ found in (piq)_2_Ir(acac). In contrast, the other two candidates show energy gaps that are only slightly higher than in the parent complex. In conclusion, our theoretical model identified (piq)_2_Ir(dend) as the triplet emitter that should suffer the least among the complexes considered from non-radiative decay and should show the highest quantum yield in photoluminescence.

Critically evaluating our predictive model, several concerns are relevant. Our model captures only the non-radiative decay based on the internal conversion of the dopant molecule to the non-radiative **T**_**2**_ state, ignoring the likely significant contributions from the other components of the device, such as the interface between the dopant and the host material. Currently, it is not at all clear which component is most important for the overall performance of the dopant. Another fundamental limitation is that we only consider one alternative triplet state in our two-state model. Although we are confident that we captured the lowest energy triplet state that should be the main contributor to the non-radiative decay, there are likely many other states within a reasonable energy range that may participate.

Moreover, the functional groups have a significant impact on the electronics and, therefore, the binding property of the acac group, which is not accounted for within our simplified conceptualization scheme. Finally, as is the case with all computer models, the accuracy may be questioned—density functional theory has been remarkably successful, but a healthy degree of skepticism is warranted. In this case, the conceptual insight of controlling the structural change by directing steric bulk in the most effective direction is qualitatively plausible and lends support to the conceptual idea even if the accuracy of the DFT based energies can be questioned. These are only some of many concerns that highlight the complicated nature of the non-radiative decay mechanisms of the OLED dopants and illustrate why it is so difficult to develop predictive models for systematically and rationally designing better dopants.

### Optical characteristics of Ir(III) complexes

**Fig. 4:**
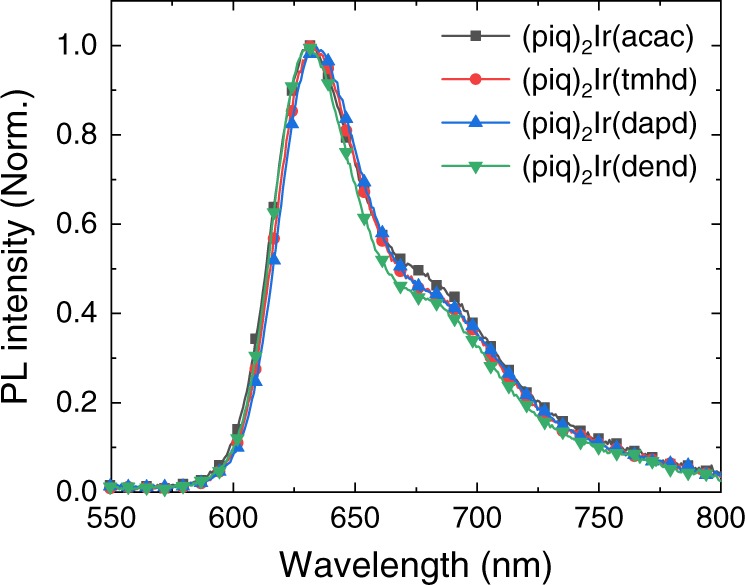
Normalized photoluminescence spectra of Ir(III) complexes doped in mixed host. The mixed host is composed with the 1:1 mixture of 9,9′-diphenyl-9H,9′H-3,3′-bicarbazole (DP-BCZ) and 5-(3-4,6-diphenyl-1,3,5-triazin-2-yl)phenyl-3,9-diphenyl-9H-carbazole (PTZP-PCZ).

The ideas that emerged from our DFT-aided conceptual model were tested by preparing all three derivatives of the parent complex and measuring their photoluminescence (PL) spectra, and the PL quantum yield using an integrating sphere. The emitters (piq)_2_Ir(acac), (piq)_2_Ir(tmhd), (piq)_2_Ir(dapd), and (piq)_2_Ir(dend) were doped at a loading of 2 wt% in the mixed host of 9,9′-diphenyl-9H,9′H-3,3′-bicarbazole (DP-BCZ) and 5-(3-4,6-diphenyl-1,3,5-triazin-2-yl)phenyl-3,9-diphenyl-9H-carbazole (PTZP-PCZ), which is the composition typically used in commercial OLED devices. The peak wavelength of the PL spectra shown in Fig. 4 varied within 3 nm depending on the ancillary ligands, as we expected. Time‐resolved PL analysis was carried out to distinguish the effects of the radiative decay rate and the non-radiative decay rate on PL quantum yield (Supplementary Fig. 1). The low radiative decay rate of 5.5 × 10^5^ s^–1^ and the relatively high non-radiative decay rate of 2.1 × 10^5^ s^–1^ seen for (piq)_2_Ir(acac) gives a relatively low PL quantum yield of only 72%. In good agreement with our predictions, the non-radiative decay rate decreased in the examined series to 1.8  × 10^5^, 1.8 × 10^5^, and 1.5 × 10^5^ s^–1^, and the PL quantum efficiencies increased to 76, 76, and 80%, as enumerated in Table 1. Thus, the tmhd ligand and the dapd ligand showed a moderately improved reduction in the non-radiative decay rate and an increase in quantum yield. The lowest non-radiative decay rate and consequently the highest PL quantum yield of 80% was achieved by (piq)_2_Ir(dend), confirming the prediction from our computer model that the 3-pentyl groups in the dend ligand are more effective than other bulky alkyl groups. Another useful feature, the full width at half maximum (FWHM) value, was narrowed from 62 nm in the parent complex to 48 nm, which likely stems from restrictions of the vibronic progression of the main chromophore by the steric demands of the ancillary ligand.

**Table 1:** Optical properties of the Ir(III) complexes.

	*λ*_max_ [nm]	FWHM [nm]	PLQY [%]	*k*_r_ [s^−1^]	*k*_nr_ [s^−1^]	Horizontal TDM [%]
(piq)_2_Ir(acac)	632	61	72	5.5 × 10^5^	2.1 × 10^5^	75
(piq)_2_Ir(tmhd)	633	53	76	5.7 × 10^5^	1.8 × 10^5^	81
(piq)_2_Ir(dapd)	634	52	76	5.6 × 10^5^	1.8 × 10^5^	73
(piq)_2_Ir(dend)	631	48	80	6.0 × 10^5^	1.5 × 10^5^	80

*PLQY* PL quantum yield, *k*_*r*_ radiative decay rate, *k*_*nr*_ non-radiative decay rate, *TDM* transition dipole moment.

The horizontal transition dipole moment was calculated by measuring angle-dependent PL spectra to confirm the effect on the efficiency of OLED devices built using these dopants (Supplementary Fig. 2). The horizontal transition dipole moments corresponded to quantum yields of 75, 81, 73, and 80%, respectively (Table 1). We note that the horizontal transition dipole moments were increased for the aliphatic substituents, but are decreased for the bulky adamantyl group. The causal connection between the structural change of the ancillary ligand and the orientation of transition dipole moment is not readily understood and is the subject of future investigations.

### Performance of Ir(III) complexes based OLEDs

**Fig. 5:**
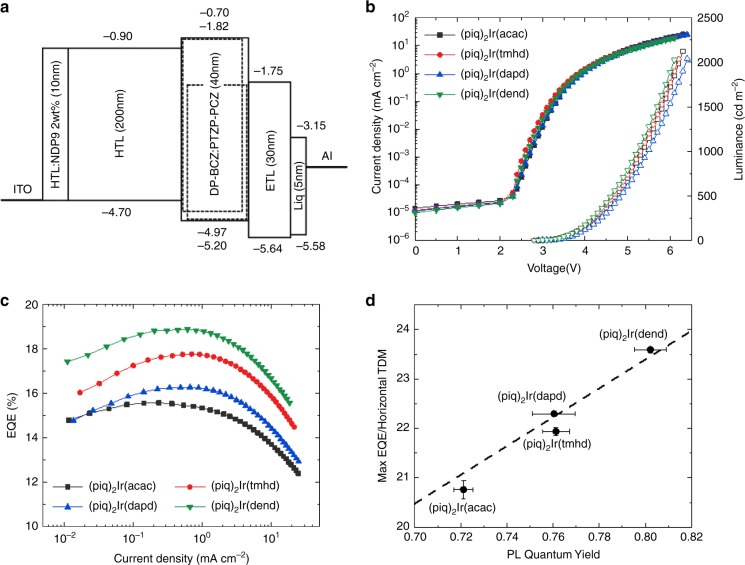
Device structure and OLED characteristics of Ir(III) complexes. **a** Schematic diagram of the OLED device structure and the energy levels of each layer. **b** The current density–voltage–luminance curves of OLEDs. **c** EQEs as a function of the current density. **d** Maximum EQE normalized by horizontal transition dipole moment according to the PL quantum yield. Error bar for PL quantum yield indicates standard error of the mean (sample standard deviation divided by the square root of 6 measurements) and error bar for *y*-axis is standard error of the mean for 4 measurements.

Finally, fully functional OLED device prototypes were fabricated to assess the effect of the increased PL quantum yield on the device performance under realistic conditions. Figure 5a illustrates the schematic configuration of the OLED device and shows the HOMO/LUMO energy level of each layer. NDP9 (Novaled GmbH) was adopted as a p-type dopant for efficient hole injection. As mentioned above, DP-BCZ and PTZP-PCZ were used at a molar ratio of 1:1 to afford a mixed host material that can easily form exciplexes and shows a good hole-electron charge balance^15^. The triplet energy level (2.45 eV) is high enough to be useful for the Ir(III) complexes. The doping concentration of the Ir(III) complexes was adjusted to be 2 wt% relative to the mixed host. Figure 5b shows the current density–voltage–luminance (*J*–*V*–*L*) characteristics of the OLEDs using the four emitters. The *J*–*V* characteristics of the OLEDs were similar, implying that the charge balance of all devices is identical. There was a difference in *L*–*V* characteristics because the electroluminescence spectra and efficiency are different. The maximum external quantum efficiencies (EQEs) of the OLED devices as a function of the current density, shown in Fig. 5c, was found to be 15.6%, 17.8%, 16.3%, and 18.9% for the four emitters, respectively. To identify the component that derives directly from the PL quantum yield, the maximum EQE must be normalized by the horizontal transition dipole moment to give the outcoupling efficiency displayed in Fig. 5d. The linear dependence of the outcoupling efficiency on the horizontal transition dipole moment confirms that the improvement of the PL quantum yield is the main source of the increased efficiency of these device prototypes. Reaching an outcoupling efficiency of 24%, the triplet emitter (piq)_2_Ir(dend) offers a substantial performance improvement over the parent molecule (piq)_2_Ir(acac).

## Discussion

A molecular mechanism for the non-radiative decay in Ir-based triplet emitter dopants for OLED devices was discovered, which is proposed to be one of the factors responsible for decreasing the photoluminescence quantum yield in triplet OLED devices. Our computational studies reveal that an alternative excited state **T**_**2**_ provides a quenching pathway for the phosphorescent **T**_**1**_ state via a thermal relaxation pathway. By inhibiting the structural distortion that gives access to this unproductive state, the quantum efficiency of photoluminescence can be significantly enhanced by nearly 10%. The improved performance is sustained in a prototype device and gave rise to an increased outcoupling efficiency of 24%. This work shows that functionalizing the ancillary ligand in a heteroleptic triplet emitter is a viable strategy for controlling the photophysical property without impacting the photoluminescent behavior of the molecule. Finally, our work shows how DFT based mechanistic explorations can be employed to implement rational and systematic optimization strategies to OLED device design.

## Methods

### Computational details

All calculations were performed using density functional theory^16^ as implemented in the Jaguar 9.1 suite of ab initio quantum chemistry program^17^. The geometries of all structures were optimized with the M06^18^ level of theory and 6-31G** basis set^19^. The singlet state is calculated with the spin multiplicity of 1, and the ^3^MLCT state is calculated with the spin multiplicity of 3 in the unrestricted calculations. The ^3^MC state is calculated using the relaxed coordinate scan by elongating the Ir–N bonds and the results are illustrated in Supplementary Fig. 3. Ir metal center was represented using the Los Alamos LACVP^19–21^ basis set. The single point energies of all optimized structures were re-evaluated using a triplet–ζ quality of basis set, cc-pVTZ(-f)^22^, where the LACV3P basis set was used for Ir. Vibrational frequencies were calculated using the optimized structures at the same level as the geometry optimizations. Calculations were repeated with other popular functionals, namely, PBE0, B3LYP, and B3LYP-D3 to benchmark the computational protocol as described in the Supplementary Discussion and these results are summarized in Supplementary Table 2.

### Optical characteristic measurements

Samples were fabricated by thermal deposition for 50-nm-thick film on fused silica substrates under a high vacuum of 10^−8^ Torr. The four Ir(III) complexes were doped at a 2 wt% level in a DP-BCZ:PTZP-PCZ host. PL spectra and PL quantum yield of the film samples were measured using Quantaurus-QY absolute PL quantum yield spectrometer (C11347-11, Hamamatsu) and excitation wavelength of 340 nm was used. Time‐resolved PL characteristics for decay time of the Ir(III) complexes were measured using time-correlated single photon counting (TCSPC) setup (Fluotime 300, PicoQuant GmbH). The samples were excited by a pulsed LED (PLS 340, PicoQuant GmbH, 0.25 MHz repetition rate at 340 nm). Angle-dependent PL spectra for calculation of transition dipole moment orientation was measured by Luxol-OLED Analyzer (CoCoLink Korea). The basic principle of this experimental equipment and the measuring method of the angle-dependent PL spectra follow the standard protocol reported previously^6,23^. The horizontal transition dipole moment was calculated using the associated fitting software.

### Device fabrication

The OLEDs were fabricated on patterned indium tin oxide (ITO) substrates. Thickness of ITO was 130 nm. ITO substrates were cleaned by isopropyl alcohol and acetone, and ultraviolet-ozone treatment was employed for 10 min. The OLEDs were fabricated under a high vacuum of 10^−8^ Torr according to the following structure: ITO (130 nm) / HIL (BCFA: NDP9 2 wt%, 10 nm) / HTL (BCFA, 200 nm) / EML (DP-BCZ: PTZP-PCZ: Ir(III) complexes 2 wt%, 40 nm) / ETL (NET-164: Liq, 1:1 wt%, 30 nm) / EIL (Liq, 5 nm) / Al (80 nm). Here BCFA, NDP9, DP-BCZ, PTZP-PCZ, NET-164 and Liq represents N-([1,1’-biphenyl]-4-yl)-9,9-dimethyl-*N*-(4-(9-phenyl-9H-carbazol-3-yl)phenyl)-9H-fluoren-2-amine, Novaled Dopand p-side (Novaled GmbH), 9,9’-diphenyl-9H,9’H-3,3’-bicarbazole, 5-(3-4,6-diphenyl-1,3,5-triazin-2-yl)phenyl-3,9-diphenyl-9H-carbazole, Novaled Electron Transporter (Novaled GmbH) and 8-quinolinolato lithium, respectively. These were used as OLED materials in the previous reports^15,24^.

### Device characteristic measurements

The current density-voltage-luminance (*J*–*V*–*L*) characteristics and EL spectra were measured using a sourcemeter (2635B, Keithley) and a spectroradiometer (SR-3AR, TOPCON). EQE were calculated based on the characteristics of *J*–*V*–*L* and the EL spectra assumed to be Lambertian surface.

## Supplementary information

Supplementary Information

## Data Availability

The authors declare that the all data supporting the findings of this study are available within the paper and the Supplementary Information files. All single point energies and DFT-optimized structures of dopants are located at Supplementary Table 1 and Supplementary Table 3.
