# Supplementary material for: Ancillary ligand increases the efficiency of heteroleptic Ir-based triplet emitters in OLED devices
Source: Nat Commun. 2020 May 8;11:2292. doi: 10.1038/s41467-020-16091-1 (PMC7210992; doi:10.1038/s41467-020-16091-1)
Supplement: Supplementary file 1 — Supplementary Information [file 41467_2020_16091_MOESM1_ESM.pdf]

Supplementary Information

Ancillary ligand increases the efficiency of heteroleptic Ir-based triplet emitters in OLED devices

Seung-yeol Baek[‡1,2], Seung-Yeon Kwak[‡3], Seoung-Tae Kim[1,2], Kyu Young Hwang[3], Hyun Koo[3], Won-Joon Son[4], Byoungki Choi[3], Sunghan Kim[3], Hyeonho Choi[3]* and Mu-Hyun Baik[2,1,]*

[1]Department of Chemistry, Korea Advanced Institute of Science and Technology (KAIST), Daejeon, 34141, Republic of Korea

[2]Center for Catalytic Hydrocarbon Functionalizations, Institute for Basic Science (IBS), Daejeon, 34141, Republic of Korea

[3]Samsung Advanced Institute of Technology (SAIT), Samsung Electronics Co., Ltd., Suwon; 16678, Republic of Korea

[4]Data and Information Technology (DIT) Center, Samsung Electronics, Hwaseong 18448, Republic of Korea

Table of contents

Supplementary Figures 2

Supplementary Tables 4

Supplementary Discussion 16

Supplementary Methods 17

Supplementary Figures

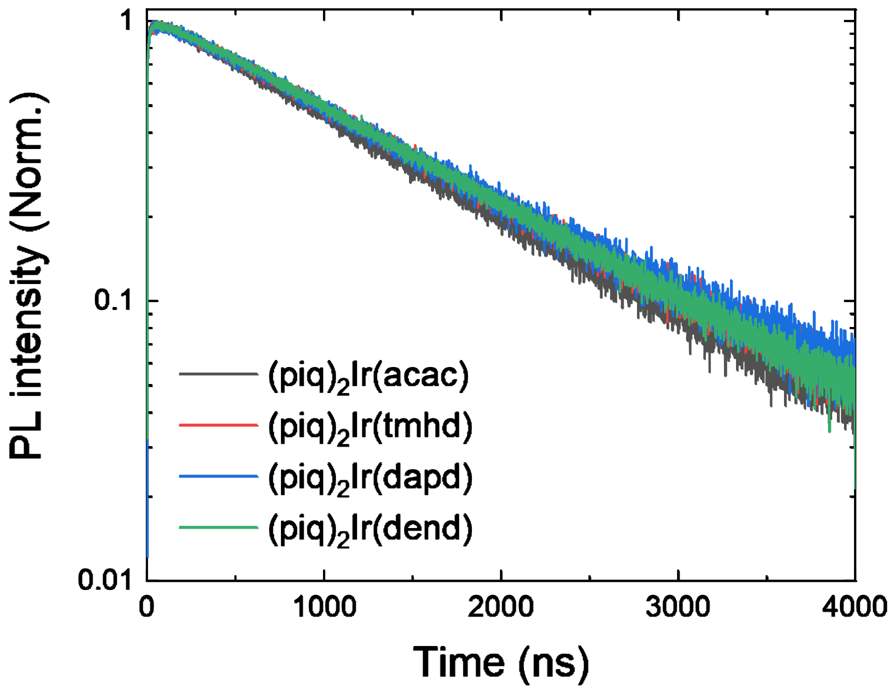

Supplementary Figure 1 | Time-resolved PL decay curves

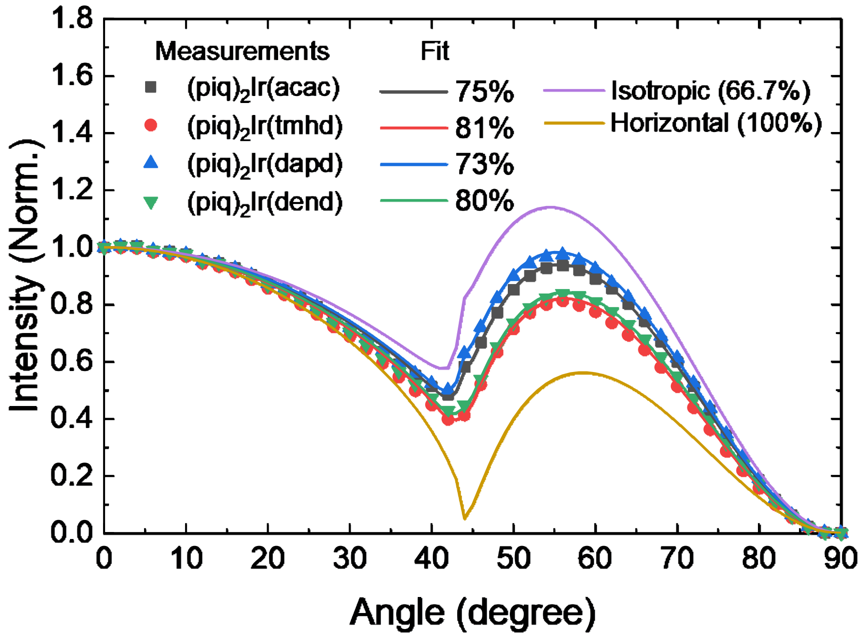

Supplementary Figure 2 | Angle-dependent PL intensities and fitting data

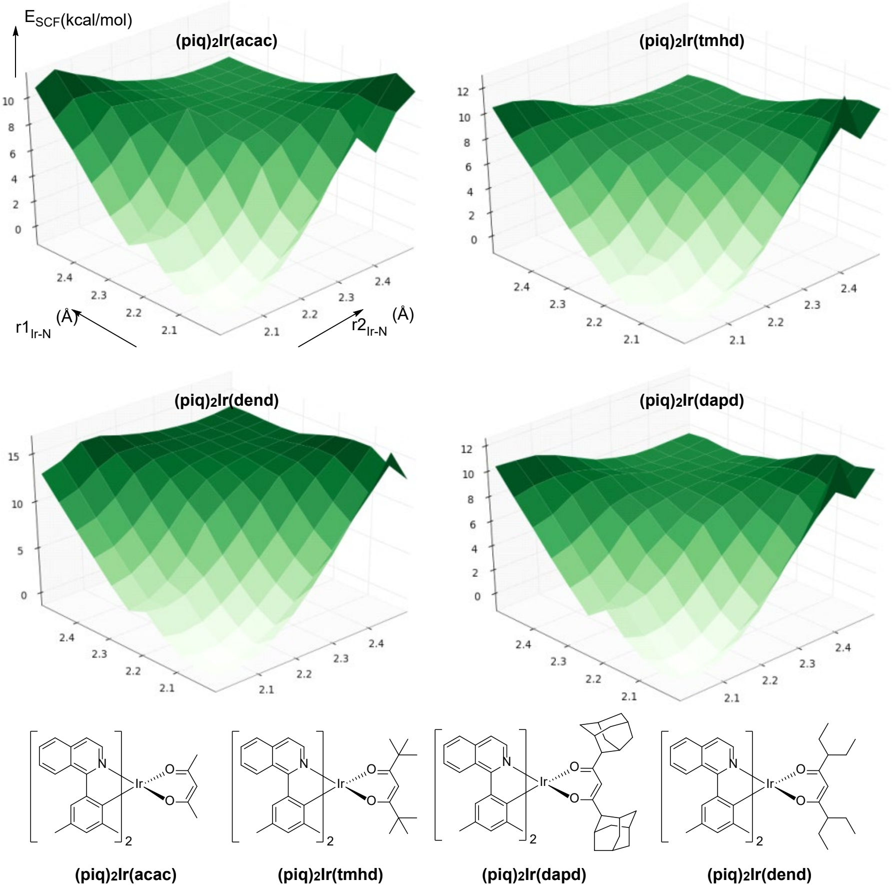

Supplementary Figure 3 | The relaxed coordinate scan results of four dopants

Supplementary Tables

DFT-optimized structure's energy components

Supplementary Table 1 | Computed energy components for DFT-optimized structures.

	E(SCF)/(eV)<br>LACVP**	E(SCF)/(eV)<br>cc-pVTZ(-f)
**acac-S_0**	-50904.508	-50917.344
**acac-T_1**	-50902.473	-50915.355
**acac-T_2**	-50902.172	-50914.902
**tmhd-T_1**	-57316.414	-57330.938
**tmhd-T_2**	-57316.051	-57330.457
**dend-T_1**	-59454.523	-59469.535
**dend-T_2**	-59453.898	-59468.84
**dapd-T_1**	-69948.523	-69965.977
**dapd-T_2**	-69948.133	-69965.508

Benchmark of functionals

Supplementary Table 2 | The calculated energy gaps of Ir(III) red dopants in various functionals

ΔE(T_2-T_1) (kcal/mol)	**(piq)$_2$Ir(acac)**	**(piq)$_2$Ir(tmhd)**	**(piq)$_2$Ir(dapd)**	**(piq)$_2$Ir(dend)**
**M06**	10.45	11.09	10.82	16.03
**B3LYP**	12.71	12.43	12.25	15.31
**B3LYP-D3**	24.05	23.31	23.25	27.28
**PBE0**	24.14	25.34	25.04	28.02

Cartesian coordinates of the optimized geometries

Supplementary Table 3 | Cartesian Coordinates of the Optimized Geometries.

==============================

acac-S0

==============================

Ir -0.215958565 0.436672270 -1.915432572
N -2.098595381 0.903702080 -2.557803392
C -2.181546926 2.149422646 -3.122958183
C -3.205173492 0.212612808 -2.211063147
C -3.444231033 2.826374292 -3.141947746
C -4.458876610 0.723508656 -2.385388136
C -4.612878323 2.073492765 -2.815419197
H -3.021486521 -0.774721503 -1.791321754
H -5.335073948 0.130780175 -2.134935856
N 1.552441597 -0.025487367 -0.989003241
C 2.124295712 0.744950533 -0.037020743
C 1.926201582 -1.323952317 -1.176595926
C 3.088302612 0.261943758 0.797771752
C 2.779122591 -1.951729059 -0.212993905
C 3.411339283 -1.123107433 0.763098419

H	1.762057066	1.770352721	0.006621360
H	3.561518669	0.911973536	1.529365897
C	0.350218594	-1.107268333	-3.037304401
C	1.389436245	-1.876250863	-2.407055140
C	-0.111302681	-1.510542631	-4.312697411
C	1.921167970	-2.997219801	-3.055277586
C	0.425097555	-2.651397705	-4.901743889
C	1.440315485	-3.400321245	-4.294996738
C	0.240271702	1.816659689	-3.269871473
C	1.489972115	2.114053249	-3.867130041
C	-0.914217591	2.587427855	-3.662986040
C	1.576933503	3.157943487	-4.780807972
C	-0.781882644	3.601808548	-4.619943619
C	0.455053270	3.902807474	-5.172672272
C	-3.605652332	4.204322338	-3.408281088
H	-2.728972912	4.830286503	-3.538555622
C	-5.876773357	2.683522940	-2.878999949
H	-6.753832340	2.082064629	-2.643075466
C	-4.859186172	4.783509731	-3.446788311
H	-4.949812412	5.847952366	-3.651847124
C	-6.006456852	4.018812180	-3.207190275
H	-1.657926559	4.128633499	-4.988904476
H	2.544774294	3.391546488	-5.227713585
C	0.594719410	4.978648186	-6.203845024
H	1.387253404	5.686814785	-5.931417942
H	0.868023872	4.559396744	-7.180954456
H	-0.335526317	5.542119503	-6.329553127
C	2.721750498	1.320718884	-3.561345100
H	2.993434668	1.379241824	-2.500956774
H	2.567711592	0.255446643	-3.782822609
H	3.573044777	1.675317287	-4.152537823
C	2.006241560	-4.603310108	-4.983023643
H	2.938848972	-4.933866024	-4.513840675
H	1.302028179	-5.445127964	-4.949461937
H	2.209695816	-4.401428223	-6.041450500
C	-1.139883280	-0.727327347	-5.066091061
H	-1.341733694	-1.181366920	-6.042331219
H	-2.085699797	-0.658816874	-4.516251087
H	-0.803364098	0.306352824	-5.229001045
H	0.061470203	-2.958552361	-5.883933544
H	2.768124819	-3.529482841	-2.631702662
C	4.298566818	-1.692664266	1.693686724
H	4.782505989	-1.036705256	2.416110754
C	2.990201235	-3.346950054	-0.126015514
H	2.430505037	-4.014092445	-0.772942781
C	3.851097345	-3.881444216	0.809946179
H	3.989289761	-4.959385395	0.855409503
C	4.532063007	-3.051773548	1.710816026
C	-1.504968643	0.644342005	1.275065422
O	-0.983870447	-0.966707826	-0.417742461
C	-1.394133568	-0.646349609	0.739290297
C	-1.168682098	1.864209533	0.667767704
O	-0.656133711	2.036883354	-0.478680342
H	-1.907771587	0.709968925	2.284805298
C	-1.419379354	3.118754625	1.464935780
H	-1.166715622	3.998043537	0.868451297
H	-2.470825672	3.176038742	1.768991828
H	-0.817809403	3.116906404	2.381865263
C	-1.845869303	-1.795174956	1.602953315
H	-2.941118717	-1.859109282	1.594634891
H	-1.439202309	-2.732557535	1.216629148
H	-1.534456134	-1.657978296	2.643668890
H	-6.991018772	4.478640079	-3.250854731
H	5.218091965	-3.479774952	2.438046217

==============================

acac-T1

==============================

Ir	-0.248487517	0.428750277	-1.859998465
N	-2.115342379	0.968415201	-2.546313524
C	-2.165945768	2.175959110	-3.149654150
C	-3.246041059	0.291987628	-2.203588486
C	-3.432992220	2.860047817	-3.235142231
C	-4.493640423	0.800525010	-2.430626392
C	-4.622510433	2.127461672	-2.918236256
H	-3.076770067	-0.675673187	-1.747142196
H	-5.376287937	0.217735067	-2.186871529
N	1.520382285	-0.094512515	-0.942636728
C	2.096616030	0.674464881	0.021747891
C	1.940185070	-1.353851438	-1.193640828
C	3.122703314	0.217295632	0.798868775
C	2.879631996	-1.968514562	-0.286382228
C	3.522029877	-1.140402555	0.690527618
H	1.684232473	1.671775341	0.115295775
H	3.590286255	0.870128095	1.529082894
C	0.293678790	-1.157257795	-2.999106407

C	1.368987918	-1.908310771	-2.424241066
C	-0.216051817	-1.614213228	-4.241414070
C	1.943218350	-3.005041361	-3.101332426
C	0.348930538	-2.736056089	-4.854955673
C	1.437147260	-3.438877821	-4.317601681
C	0.261087239	1.848787546	-3.215684414
C	1.524943590	2.195063591	-3.758665085
C	-0.865732193	2.607849121	-3.666495562
C	1.618438959	3.219359398	-4.705797672
C	-0.736268759	3.594821453	-4.666080952
C	0.504685819	3.920588017	-5.191084385
C	-3.567501783	4.243131161	-3.533250570
H	-2.680444717	4.850432873	-3.649896145
C	-5.887564182	2.754292727	-3.043420076
H	-6.779447556	2.172899246	-2.824080229
C	-4.809727669	4.835765839	-3.616128683
H	-4.885557175	5.897149086	-3.833608866
C	-5.982980251	4.079975128	-3.401065826
H	-1.614178777	4.072799683	-5.082571030
H	2.600774527	3.464255095	-5.108141422
C	0.653930485	4.962255955	-6.276175022
H	1.431276798	5.694028378	-6.026525497
H	0.936932385	4.509990692	-7.235102654
H	-0.280077666	5.510594368	-6.434133530
C	2.799404860	1.487008572	-3.356739044
H	3.087374449	1.727070212	-2.327174187
H	2.687892914	0.401909083	-3.417554617
H	3.628959179	1.782523394	-4.007262230
C	2.042070150	-4.613502502	-5.050548077
H	3.014621973	-4.887807369	-4.629905701
H	1.397520661	-5.500176907	-4.993099689
H	2.188183784	-4.392820835	-6.113967419
C	-1.353580117	-0.918000638	-4.954969883
H	-1.479004741	-1.317127585	-5.966747284
H	-2.304619312	-1.053052902	-4.427940845
H	-1.175995231	0.157166854	-5.032509327
H	-0.054359727	-3.058901548	-5.814082146
H	2.834547281	-3.483261585	-2.714448690
C	4.496182442	-1.699115276	1.555875659
H	4.989623070	-1.046250463	2.271336079
C	3.151751518	-3.362930059	-0.249055400
H	2.587643862	-4.032098293	-0.883973837
C	4.082629204	-3.884725571	0.624467015
H	4.263976574	-4.955450535	0.638301313
C	4.787296772	-3.043983459	1.514192581
C	-1.593161702	0.645755827	1.310976624
O	-1.076375842	-0.976715386	-0.388891727
C	-1.507673025	-0.649652123	0.765439510
C	-1.199078441	1.864665627	0.725343883
O	-0.653534114	2.034415007	-0.414160907
H	-2.016074419	0.713768065	2.308941603
C	-1.424945235	3.135470390	1.531895638
H	-1.115268111	4.002851486	0.946789742
H	-2.481845379	3.239720106	1.801011562
H	-0.853265405	3.108210325	2.466746807
C	-2.007925510	-1.806007624	1.618435860
H	-3.103061676	-1.791069984	1.675451517
H	-1.692087531	-2.752087831	1.175738454
H	-1.627338529	-1.735172272	2.642394781
H	-6.956630707	4.554744720	-3.484720469
H	5.529620647	-3.464551687	2.186469316

==============================

acac-T2

==============================

Ir	-0.471191615	0.405701190	-1.408875108
N	-2.736956835	0.457516938	-2.174456120
C	-2.894784451	1.697529912	-2.651994228
C	-3.761390924	-0.187671766	-1.562116861
C	-4.123423100	2.414544106	-2.439404726
C	-5.009618282	0.361071765	-1.434587479
C	-5.221374512	1.700515389	-1.854548097
H	-3.529959917	-1.184210896	-1.196279526
H	-5.822344303	-0.205414727	-0.988881111
N	1.642330527	0.415491939	-0.326875687
C	1.929844141	1.173036218	0.761952937
C	2.092804193	-0.841844857	-0.427770585
C	2.723014593	0.725819886	1.784371734
C	2.811666250	-1.451689839	0.659760833
C	3.174304962	-0.620785296	1.771102190
H	1.503214002	2.172407389	0.757481098
H	2.980408430	1.378819704	2.613584995
C	0.538465977	-1.069531679	-2.362963915
C	1.737408996	-1.497716784	-1.706451297
C	0.204354703	-1.713344216	-3.585925341
C	2.588187933	-2.443415880	-2.296519756

C 1.053074479 -2.690513372 -4.116973877
C 2.258237839 -3.056985140 -3.505409241
C -0.412068516 1.749001741 -2.920606852
C 0.725560784 2.265515804 -3.604195356
C -1.696969867 2.221272707 -3.348757505
C 0.558138371 3.165950060 -4.661108494
C -1.825733304 3.084512711 -4.444656849
C -0.702652156 3.572044373 -5.114305496
C -4.303419590 3.798261881 -2.715948582
H -3.463598013 4.381457329 -3.069377899
C -6.464784145 2.355810404 -1.668009996
H -7.290768623 1.792735100 -1.240698695
C -5.518153667 4.413845062 -2.504237175
H -5.628805637 5.473335266 -2.717592001
C -6.616914749 3.683788776 -1.997388482
H -2.812180758 3.353480816 -4.809676647
H 1.443936229 3.552226305 -5.163047314
C -0.839449286 4.493833065 -6.302339077
H -0.226168826 5.394423962 -6.181286335
H -0.509109020 4.004837036 -7.227235794
H -1.876748204 4.810045242 -6.446743011
C 2.128701210 1.853074551 -3.237148046
H 2.314212084 1.978071332 -2.168322325
H 2.291679144 0.791221321 -3.451967001
H 2.867665529 2.433995724 -3.797707081
C 3.166894913 -4.087018490 -4.131999969
H 4.152766228 -4.090540886 -3.657416821
H 2.749931335 -5.097723007 -4.035312653
H 3.307941437 -3.900378704 -5.202343464
C -1.036086440 -1.344588280 -4.361331940
H -1.203745604 -2.040605307 -5.189191818
H -1.919584870 -1.334124923 -3.719751120
H -0.949239254 -0.334013969 -4.776931286
H 0.775112569 -3.169411659 -5.054745674
H 3.541613102 -2.677425385 -1.833279729
C 3.925188065 -1.169186354 2.841190815
H 4.207626343 -0.519132614 3.665675879
C 3.135831594 -2.835507870 0.731632769
H 2.789158344 -3.501996756 -0.047169425
C 3.849472284 -3.344952106 1.795604587
H 4.080642223 -4.405827522 1.830365181
C 4.269112587 -2.502468348 2.849654198
C -1.921103239 0.504842460 1.765753150
O -0.822088778 -0.972416818 0.233791426
C -1.401695728 -0.731654823 1.343024254
C -1.874514341 1.717219949 1.054543734
O -1.374539971 1.894191146 -0.103941277
H -2.383193254 0.531551361 2.747729540
C -2.454148293 2.958760023 1.712646961
H -2.496737480 3.775134087 0.989934564
H -3.459920168 2.761758327 2.098291397
H -1.833350658 3.266839981 2.562314272
C -1.518725157 -1.916935325 2.285675526
H -2.553590298 -2.047332048 2.620943546
H -1.180591464 -2.824699640 1.783818603
H -0.906262696 -1.753399014 3.179409504
H -7.572324276 4.178390026 -1.845390320
H 4.840494633 -2.913594007 3.677342415

===============================

tmhd-T1

===============================

Ir -0.208005354 0.432797015 -1.918598652
N -2.099717379 0.931000113 -2.553617716
C -2.168981552 2.179681301 -3.124975443
C -3.218902826 0.260466993 -2.194161415
C -3.426259995 2.881504059 -3.131920815
C -4.470717430 0.792939484 -2.354278803
C -4.611485004 2.147083998 -2.787279367
H -3.052415848 -0.722777843 -1.769103408
H -5.348437786 0.211360827 -2.091331244
N 1.570944667 -0.064890690 -0.998165190
C 2.150961161 0.677514493 -0.024404684
C 1.944765210 -1.363883018 -1.218362927
C 3.119162083 0.169983685 0.799431980
C 2.803694725 -2.022390127 -0.269904792
C 3.444635153 -1.217813015 0.731835425
H 1.789844632 1.696661234 0.055888761
H 3.590024948 0.804650903 1.543334126
C 0.320296139 -1.134303212 -3.054797173
C 1.390919805 -1.895878911 -2.457385778
C -0.196997747 -1.570624828 -4.305079460
C 1.926254153 -3.007958651 -3.129902124
C 0.338439345 -2.710032463 -4.910746574
C 1.402879477 -3.432889938 -4.348116398
C 0.265808702 1.833266020 -3.268393278

C	1.526326060	2.164599419	-3.838161230
C	-0.891979992	2.601881742	-3.675487757
C	1.611960292	3.209761858	-4.759689808
C	-0.758721769	3.615507841	-4.641864777
C	0.483333230	3.935508728	-5.181750298
C	-3.569532156	4.268322468	-3.397651672
H	-2.688517094	4.879074574	-3.543143272
C	-5.869765282	2.783376694	-2.836624861
H	-6.755331039	2.202748060	-2.590618372
C	-4.817740917	4.871776104	-3.420689106
H	-4.892139912	5.936102390	-3.625927687
C	-5.979045868	4.125656128	-3.164053917
H	-1.635185719	4.125765800	-5.021209240
H	2.583179474	3.458211184	-5.182850361
C	0.619623363	5.015491009	-6.226644516
H	0.932437241	4.596706390	-7.191529751
H	-0.323474258	5.546782970	-6.381999969
H	1.380283475	5.751055717	-5.939013958
C	2.786735058	1.407221437	-3.499948502
H	3.081847191	1.559671402	-2.457340479
H	2.650348902	0.330182225	-3.634647846
H	3.615628719	1.732274652	-4.135803223
C	1.974010825	-4.634607792	-5.059904099
H	2.891953707	-4.986045837	-4.580166340
H	1.259670973	-5.467686176	-5.063246727
H	2.203637838	-4.407292366	-6.107191086
C	-1.293317914	-0.829706311	-5.029982090
H	-1.460278988	-1.260002136	-6.022039413
H	-2.238996983	-0.868603170	-4.481564522
H	-1.043363690	0.228557691	-5.151035309
H	-0.068682827	-3.034659147	-5.866341114
H	2.794843674	-3.518918514	-2.734749317
C	4.335240364	-1.818976879	1.647861123
H	4.824212074	-1.188979268	2.386643648
C	3.010971546	-3.426650524	-0.217046976
H	2.451647520	-4.074086189	-0.879022419
C	3.875933170	-3.991910934	0.705931604
H	4.010054111	-5.069601536	0.723683000
C	4.562848091	-3.184449196	1.629438162
C	-1.547826529	0.658739150	1.288104773
O	-0.986742496	-0.939919829	-0.400608867
C	-1.407401204	-0.641087055	0.766705751
C	-1.203922749	1.877676249	0.673737228
O	-0.640961230	2.010326147	-0.463398814
H	-1.971453905	0.730023861	2.281381845
C	-1.508714676	3.199915886	1.427098274
C	-1.796426296	-1.844962120	1.666108727
H	-6.954660892	4.602623940	-3.197802305
H	5.249773979	-3.632152319	2.342261314
C	-3.273107052	-1.714807272	2.102269173
H	-3.939345837	-1.675153136	1.233719230
H	-3.561537743	-2.583077908	2.706219435
H	-3.451145887	-0.817575216	2.702142239
C	-1.611876369	-3.167757988	0.902396977
H	-0.577016115	-3.300457478	0.578850091
H	-1.885033011	-4.007153511	1.551666737
H	-2.242918730	-3.206446409	0.009795159
C	-0.882711768	-1.857430696	2.914806128
H	-1.001792550	-0.955108225	3.521066666
H	-1.124605536	-2.720763683	3.546205521
H	0.171233580	-1.934638500	2.628307819
C	-3.017623186	3.264667034	1.763942599
H	-3.626068354	3.206915140	0.855455160
H	-3.329845428	2.455236197	2.430236816
H	-3.246772528	4.213986397	2.263029337
C	-1.144095778	4.412469864	0.551713943
H	-0.083719425	4.413406372	0.285478950
H	-1.717221856	4.417583942	-0.378889859
H	-1.362650871	5.337363243	1.097416401
C	-0.682859182	3.250016451	2.731994867
H	-0.880102456	4.188974380	3.262593508
H	-0.928581059	2.425274134	3.407186985
H	0.391054988	3.204372168	2.520737171

==============================

tmhd-T2

==============================

Ir	-0.482477039	0.445426017	-1.448190212
N	-2.715403080	0.462251961	-2.232618332
C	-2.911967039	1.667720318	-2.780221462
C	-3.731760979	-0.212439701	-1.639361024
C	-4.188978195	2.322034359	-2.659224272
C	-5.013739109	0.266802043	-1.604467154
C	-5.275710106	1.572020888	-2.100026846
H	-3.462086439	-1.173447847	-1.210042596
H	-5.816839218	-0.324366957	-1.173560858

N	1.658805728	0.370574862	-0.333401799
C	1.977983594	1.066806316	0.784917593
C	2.086408854	-0.884112120	-0.505091190
C	2.788831234	0.559498012	1.766039610
C	2.815846205	-1.560798645	0.534910977
C	3.217560291	-0.791122973	1.677077413
H	1.568145752	2.071672678	0.840284944
H	3.076622963	1.168252349	2.618573427
C	0.507831931	-1.011989474	-2.443654537
C	1.712723136	-1.470622301	-1.813984275
C	0.170609191	-1.606043816	-3.694792271
C	2.565731287	-2.378606796	-2.454891443
C	1.020646334	-2.553736687	-4.274608135
C	2.233505964	-2.937119722	-3.690232754
C	-0.432381570	1.804664373	-2.959697247
C	0.710430741	2.382647276	-3.576556683
C	-1.712907314	2.222646236	-3.443682432
C	0.555779994	3.269057989	-4.647565842
C	-1.826271057	3.071164370	-4.557490349
C	-0.697789431	3.606797934	-5.175103664
C	-4.428786278	3.681946278	-3.001614332
H	-3.604423523	4.299632072	-3.333075047
C	-6.561772823	2.161776781	-2.007952690
H	-7.377257347	1.570003152	-1.599545240
C	-5.685729027	4.235523701	-2.879592657
H	-5.841148853	5.278454304	-3.141014338
C	-6.768883228	3.464746714	-2.401955605
H	-2.804157972	3.285879612	-4.976660252
H	1.447610259	3.697433710	-5.102857113
C	-0.817161620	4.515366554	-6.376043797
H	-0.290498942	4.103596687	-7.245429516
H	-1.863011956	4.665616035	-6.660512447
H	-0.380450040	5.501668930	-6.176318169
C	2.110481501	2.049959898	-3.120011091
H	2.230158567	2.219448805	-2.047076225
H	2.340410471	0.992617846	-3.289881229
H	2.850988388	2.652657032	-3.655373573
C	3.147488832	-3.927412987	-4.369514465
H	4.142879009	-3.928407192	-3.915369749
H	2.750347614	-4.948135853	-4.297501564
H	3.261358500	-3.702603102	-5.435550690
C	-1.085337400	-1.232417583	-4.442075729
H	-1.198064208	-1.839816570	-5.345570087

H	-1.973663449	-1.361900568	-3.819352627
H	-1.068327427	-0.177544892	-4.734959126
H	0.738873422	-2.991791248	-5.230715275
H	3.523190260	-2.627584457	-2.009152889
C	3.985955954	-1.403264999	2.699970245
H	4.297704220	-0.799397230	3.548647881
C	3.124285221	-2.950416327	0.528242886
H	2.753648043	-3.571574211	-0.277128905
C	3.855510473	-3.522674799	1.547758341
H	4.072055817	-4.586667538	1.523026347
C	4.311201572	-2.739224672	2.632735491
C	-1.737107158	0.738221228	1.797859311
O	-0.885692120	-0.856217206	0.242080063
C	-1.333836198	-0.549340606	1.394785523
C	-1.759812236	1.904560804	1.009757400
O	-1.366444826	1.987437010	-0.200449601
H	-2.106714964	0.832592964	2.810079813
C	-2.315036535	3.213795900	1.633734822
C	-1.445561051	-1.715913057	2.413041592
H	-7.756962299	3.909397602	-2.320947170
H	4.896603584	-3.197903872	3.424919605
C	-2.925695896	-1.906460047	2.819358587
H	-3.548502922	-2.133493900	1.947076917
H	-3.016244411	-2.742887020	3.522216797
H	-3.338817358	-1.016926050	3.303272963
C	-0.923963189	-3.023985624	1.793509483
H	0.127111569	-2.938736916	1.506216884
H	-1.017063260	-3.837496281	2.522225857
H	-1.489203215	-3.296864510	0.898251235
C	-0.604071081	-1.384045601	3.668128014
H	-0.962391853	-0.486235052	4.180632114
H	-0.652453482	-2.215833187	4.380931854
H	0.447185636	-1.225164413	3.406581402
C	-3.776746035	2.990720034	2.087545633
H	-4.407717228	2.683743954	1.246954679
H	-3.855665445	2.224975586	2.865175962
H	-4.187376499	3.922565222	2.494330645
C	-2.281264544	4.357315540	0.605079591
H	-1.265931606	4.542234898	0.244512096
H	-2.903254747	4.131105900	-0.264390439
H	-2.657255173	5.277163029	1.067862749
C	-1.450825453	3.611581802	2.853859186
H	-1.829100370	4.541831017	3.293834448

H -1.459489107 2.845920324 3.634781122
H -0.408699870 3.781078100 2.560953617

==============================
dend-T1
==============================

Ir -0.233554274 0.432394713 -1.863656163
N -2.114723921 0.945744872 -2.518272400
C -2.174134016 2.169122934 -3.139770269
C -3.239871502 0.266776294 -2.196177483
C -3.441313744 2.846583843 -3.240028143
C -4.490937233 0.767809927 -2.445132256
C -4.630965710 2.101415634 -2.938449144
H -3.079767704 -0.700359643 -1.734769702
H -5.370852470 0.177255496 -2.211241245
N 1.560666800 -0.074446172 -0.985310435
C 2.185609102 0.684455335 -0.054029830
C 1.968445897 -1.354344964 -1.251150608
C 3.239936352 0.213013425 0.682335317
C 2.926190376 -1.983429670 -0.378668189
C 3.620002031 -1.158330441 0.569585919
H 1.796434760 1.689327598 0.058856890
H 3.744603634 0.863340437 1.389393926
C 0.270664573 -1.127107978 -3.024342060
C 1.370941162 -1.883028507 -2.470345259
C -0.273308575 -1.553727865 -4.268020630
C 1.898940563 -2.980304718 -3.174443960
C 0.257235140 -2.679659367 -4.902547359
C 1.346077800 -3.396944523 -4.381519794
C 0.264888287 1.821433902 -3.221148968
C 1.533070564 2.134411812 -3.786674738
C -0.884303033 2.582501411 -3.665957689
C 1.633073568 3.157447577 -4.732163429
C -0.735218167 3.571031094 -4.656630039
C 0.514031827 3.876968145 -5.186779499
C -3.594662905 4.219390392 -3.565167189
H -2.719100714 4.843882084 -3.678098440
C -5.895575047 2.709269524 -3.086409569
H -6.783202171 2.119713783 -2.870394230
C -4.849673271 4.795679569 -3.685334921
H -4.930620670 5.850497246 -3.933492184
C -6.010458469 4.035247326 -3.471846819
H -1.605231166 4.070619106 -5.062988758
H 2.610392570 3.389730692 -5.150290012
C 0.669930339 4.935554981 -6.250683784
H 1.375906467 5.713166714 -5.933932781
H 1.064406395 4.509062767 -7.181198120
H -0.283515364 5.419829369 -6.479444504
C 2.792273760 1.381316781 -3.432904243
H 3.093051434 1.554448485 -2.396142244
H 2.654886246 0.302006900 -3.547897339
H 3.619302273 1.691444516 -4.078423977
C 1.915144682 -4.575320244 -5.133365631
H 2.782845736 -4.999268055 -4.620122433
H 1.168227792 -5.371080875 -5.244162083
H 2.227446079 -4.289480209 -6.145116806
C -1.392221928 -0.819618642 -4.965914249
H -1.563245893 -1.238006830 -5.962320805
H -2.331301928 -0.883740544 -4.409687042
H -1.161109686 0.244049549 -5.076724052
H -0.173943087 -2.996440172 -5.850148678
H 2.785582304 -3.484570265 -2.813885212
C 4.617864132 -1.723102093 1.394013166
H 5.143141747 -1.074459076 2.090729952
C 3.196521044 -3.377233028 -0.353204906
H 2.604614019 -4.048285961 -0.961198807
C 4.168379784 -3.906582832 0.480373859
H 4.347989082 -4.978188992 0.475805074
C 4.907093048 -3.076267481 1.341101766
C -1.552104235 0.661015093 1.377808928
O -1.040215254 -0.936701953 -0.333916128
C -1.459393620 -0.633839011 0.835770667
C -1.172093153 1.874575019 0.775683701
O -0.633647501 2.011432648 -0.375819892
H -1.964188933 0.732149124 2.378213406
C -1.406445503 3.183766365 1.546503425
H -1.038444519 3.974960089 0.881099582
C -1.894144535 -1.826814771 1.702521801
H -1.744953871 -2.709286451 1.067624450
H -6.990770817 4.490759850 -3.579551935
H 5.675988674 -3.497724771 1.982067466
C -3.384378195 -1.770192027 2.113416910
H -3.587038040 -2.654810667 2.731957674
H -3.550665855 -0.903307319 2.766487598
C -4.377796650 -1.731181741 0.949218512
H -4.273198605 -0.811399341 0.365344942

H	-4.230815411	-2.580922127	0.272025049
H	-5.409214973	-1.774202704	1.315944076
C	-1.012943029	-1.983101964	2.966147661
H	-1.176359534	-1.122109175	3.627970457
H	-1.381331801	-2.859510899	3.516287088
C	0.484587580	-2.146474838	2.695695400
H	0.907882333	-1.256052256	2.221549034
H	1.031133533	-2.314261198	3.630120277
H	0.683853805	-2.998578310	2.036951303
C	-2.907552004	3.441705465	1.824502349
H	-3.283346415	2.675829887	2.516099691
H	-2.981328726	4.395679951	2.364021063
C	-3.797726154	3.492425203	0.580898225
H	-3.815181017	2.531666517	0.058102477
H	-4.829752922	3.738774300	0.853217781
H	-3.452625036	4.250099659	-0.130907074
C	-0.613392830	3.256024122	2.872562170
H	-0.849899352	4.219525814	3.343617916
H	-0.983812869	2.485803127	3.561930895
C	0.904948413	3.124894142	2.727286816
H	1.402766943	3.267390966	3.692656279
H	1.186056495	2.135238886	2.353911161
H	1.304225802	3.874474525	2.033877850

==============================

dend-T2

==============================

Ir	-0.168503210	0.403829128	-2.091873884
N	-2.401916265	0.802287400	-2.896973372
C	-2.397081137	2.073294401	-3.311770439
C	-3.525808811	0.207201406	-2.434693575
C	-3.565021515	2.889898777	-3.115959644
C	-4.731096745	0.855829239	-2.388592243
C	-4.768722534	2.244174242	-2.688271523
H	-3.405615807	-0.824823499	-2.120621204
H	-5.629477024	0.340241283	-2.062300205
N	1.970691442	0.047786705	-1.064921021
C	2.468899488	0.756425381	-0.026106101
C	2.227057219	-1.255059719	-1.220495343
C	3.292406321	0.195581079	0.913581371
C	2.920209169	-1.968967319	-0.182638526
C	3.504971743	-1.208755255	0.880281091
H	2.183142662	1.803054214	-0.004161119
H	3.723140240	0.797580838	1.708135366
C	0.640503645	-1.165493965	-3.131326914
C	1.732762933	-1.818071842	-2.494193792
C	0.219356909	-1.664256454	-4.386377811
C	2.412526369	-2.878249168	-3.123133898
C	0.891450703	-2.750502110	-4.958086491
C	1.999296904	-3.363834620	-4.357974529
C	0.061241593	1.805649161	-3.567607641
C	1.259316802	2.161541939	-4.230990887
C	-1.127143860	2.495627403	-3.936904669
C	1.232392192	3.150756836	-5.220339298
C	-1.121099353	3.452227354	-4.969019890
C	0.056084361	3.798258066	-5.619943619
C	-3.574053049	4.307288170	-3.219947100
H	-2.652925730	4.826282024	-3.453990459
C	-5.936978340	3.015680790	-2.477180481
H	-6.842206001	2.503584385	-2.158688784
C	-4.718797207	5.028934002	-2.964555264
H	-4.685072422	6.113368988	-3.023098707
C	-5.934241295	4.389295101	-2.600676537
H	-2.055501938	3.892284155	-5.301780701
H	2.166052818	3.414614916	-5.715788364
C	0.074265614	4.820677757	-6.732680321
H	0.716644287	5.673670769	-6.480944633
H	0.461316794	4.392775059	-7.665405750
H	-0.928741574	5.209211826	-6.935902596
C	2.576690435	1.497566342	-3.901363373
H	2.847177267	1.656958461	-2.853664637
H	2.516673326	0.415137827	-4.043650627
H	3.384085894	1.886163235	-4.530145645
C	2.718949556	-4.502201080	-5.043310165
H	3.610888243	-4.806057453	-4.486232758
H	2.071761608	-5.383080482	-5.139516830
H	3.035461903	-4.225612164	-6.056083202
C	-0.942068636	-1.049730420	-5.133406162
H	-1.105229020	-1.549990296	-6.093379498
H	-1.864381790	-1.114022851	-4.548760414
H	-0.769330263	0.013580229	-5.320747852
H	0.549911797	-3.122569084	-5.923459053
H	3.305576563	-3.292603016	-2.665513277
C	4.198301792	-1.878124952	1.918068409
H	4.640875340	-1.280164003	2.712032080
C	2.980889320	-3.385936737	-0.093213163

H 2.465923309 -3.984770536 -0.834234416
C 3.633045673 -4.003816128 0.950272799
H 3.640049458 -5.089107037 1.001570106
C 4.274033546 -3.254284143 1.973219633
C -1.435544133 0.599883378 1.119942307
O -0.827320695 -1.011857271 -0.554592133
C -1.253208041 -0.696943104 0.608777702
C -1.177529812 1.828635335 0.486984611
O -0.689078450 2.002348661 -0.681417704
H -1.832376719 0.660118461 2.126750469
C -1.492493749 3.116275787 1.260469913
H -1.241832137 3.931612492 0.570599914
C -1.612667441 -1.883217216 1.511684418
H -1.342770338 -2.774051189 0.930975556
C -3.127869844 -1.935432076 1.817866921
H -3.319809675 -2.857065201 2.383331776
H -3.386322021 -1.106101036 2.489760160
C -4.029130936 -1.885477543 0.582239270
H -3.954311132 -0.912840724 0.087401912
H -3.749187708 -2.656362772 -0.145164967
H -5.078479290 -2.042749405 0.854245007
C -0.813564539 -1.894320726 2.835004330
H -1.142507672 -1.055176497 3.462588072
H -1.089608073 -2.806056976 3.382025242
C 0.703145862 -1.833513618 2.655466557
H 1.004098058 -0.896608353 2.178636312
H 1.219494939 -1.895739198 3.619700193
H 1.067917228 -2.652768135 2.028150320
C -2.986117840 3.232341766 1.641974211
H -3.228207111 2.463007450 2.387494564
H -3.125530720 4.197412968 2.147896051
C -3.949143887 3.120835066 0.460760236
H -3.875494003 2.138478518 -0.013982537
H -4.987960339 3.256959200 0.781821549
H -3.735347986 3.872660875 -0.305275291
C -0.623940825 3.260848761 2.532519579
H -0.839025140 4.245280743 2.969035864
H -0.949583232 2.521162987 3.276061535
C 0.880654871 3.113598585 2.299539804
H 1.444276929 3.349541187 3.208674669
H 1.128106475 2.088624716 2.009852409
H 1.227220893 3.783244133 1.503773928
H 4.806192875 -3.748790979 2.772387743
H -6.832034588 4.963463306 -2.425468445

================================

dapd-T1

================================

Ir -0.204363093 0.390039533 -1.970016718
N -2.069350004 0.978383064 -2.614760399
C -2.075876474 2.245914459 -3.158154249
C -3.222533464 0.358504772 -2.278176546
C -3.296254873 3.009650707 -3.154832363
C -4.446625233 0.956295729 -2.435550451
C -4.518525600 2.327426910 -2.835584641
H -3.110074043 -0.640231431 -1.871247530
H -5.353435993 0.413601488 -2.188494682
N 1.550887942 -0.188839793 -1.052935839
C 2.109793901 0.497212023 -0.025980093
C 1.922558069 -1.470626473 -1.340228438
C 3.062722683 -0.056199431 0.784665525
C 2.775331259 -2.178848982 -0.423796564
C 3.396067858 -1.434305668 0.635760546
H 1.744385719 1.509430766 0.105381101
H 3.516690731 0.533223987 1.574841857
C 0.302793026 -1.146029592 -3.165072680
C 1.368615866 -1.940120459 -2.607803822
C -0.218598127 -1.521628022 -4.431721687
C 1.901372910 -3.020512581 -3.332765341
C 0.309052497 -2.636593580 -5.089601994
C 1.371023536 -3.390223742 -4.567092896
C 0.345611751 1.811772585 -3.265046597
C 1.630063415 2.118902206 -3.796475172
C -0.778466225 2.628213167 -3.679028988
C 1.769325972 3.181945324 -4.689624786
C -0.590561509 3.659326077 -4.618432045
C 0.672877669 3.951300859 -5.121263027
C -3.367202520 4.408766747 -3.381844282
H -2.455593586 4.977746964 -3.507205486
C -5.741001606 3.029151917 -2.871010542
H -6.656199455 2.488463640 -2.643914700
C -4.583481312 5.076799870 -3.393015623
H -4.601539612 6.148839951 -3.570541143
C -5.781647205 4.384764671 -3.161923885
H -1.441450000 4.207841873 -5.002812862
H 2.757655859 3.411948204 -5.082244396

C 0.868160248 5.046921730 -6.139747143
H 1.696288347 5.707275391 -5.857307434
H 1.114501715 4.631338596 -7.125224113
H -0.031565644 5.658585548 -6.251074791
C 2.863126516 1.323271751 -3.445852518
H 3.128274679 1.433025837 -2.390098095
H 2.711576223 0.254849076 -3.625130177
H 3.717911482 1.653527021 -4.043663979
C 1.923925996 -4.569836617 -5.328225613
H 2.894356966 -4.883101463 -4.932285309
H 1.246810555 -5.431576729 -5.264545441
H 2.048181295 -4.338821411 -6.391830444
C -1.312311292 -0.743104815 -5.121121407
H -1.495481491 -1.139135003 -6.124603271
H -2.252675295 -0.785523832 -4.564069271
H -1.048845768 0.315122306 -5.211817265
H -0.104026355 -2.914459705 -6.057452202
H 2.771749258 -3.549384594 -2.964876652
C 4.274929047 -2.086057186 1.529379249
H 4.747067928 -1.499857187 2.313892603
C 2.993794680 -3.582418680 -0.456102759
H 2.451204777 -4.192880154 -1.163677216
C 3.844252348 -4.196435928 0.446014404
H 3.984758615 -5.272861958 0.399751127
C 4.511069775 -3.445070028 1.430593848
C -1.645307422 0.554345727 1.198690295
O -1.021586537 -1.009035110 -0.497702718
C -1.456667900 -0.737182796 0.672545910
C -1.291078806 1.785663128 0.613220334
O -0.639885008 1.932014108 -0.475902617
H -2.118347168 0.607650280 2.171169519
H -6.731632710 4.911478519 -3.183013439
H 5.185156822 -3.934835434 2.127658844
C -2.780415058 -3.097327471 4.135966301
C -1.282483578 -3.127532482 3.766030073
C -0.965985656 -4.435025215 3.010772467
H -0.675220191 -3.068491697 4.679092884
C -0.952684999 -1.917792797 2.866510153
C -3.632736206 -3.168789625 2.850802898
H -3.014018774 -2.180383921 4.694950581
H -3.023442030 -3.941000938 4.795887470
C -3.313645601 -4.477440834 2.098478317
H -4.698579788 -3.141735077 3.113697052
C -3.308152199 -1.957459807 1.949631453
C -1.816802144 -4.497046947 1.724068880
H -3.931622505 -4.553849220 1.193506360
H -3.559993744 -5.343703747 2.727405787
H -1.586126447 -5.419370651 1.175176263
C -1.490150809 -3.286248922 0.823547184
H 0.102576375 -4.477416992 2.760885000
H -1.180990100 -5.302377224 3.649762392
H -1.142635345 -0.987694144 3.415370703
H 0.114288822 -1.918334246 2.607564449
C -1.801407337 -1.957518816 1.559759736
H -0.434793651 -3.306962967 0.530103922
H -2.069379807 -3.338956118 -0.105391562
H -3.568126678 -1.028965712 2.472542048
H -3.921360254 -1.989725828 1.038876772
C -1.712985277 3.092909336 1.323396325
C -3.722715855 4.493409157 2.032457352
C -3.210439920 4.497697353 3.488720894
C -1.667558432 4.428149223 3.492537737
H -3.630260229 3.645451546 4.041266918
H -3.547173500 5.407068253 4.004531384
C -3.149558544 5.712934494 1.280545235
H -4.819981098 4.535068035 2.026719093
C -3.268465757 3.194734573 1.331708074
C -1.607868433 5.634405136 1.285103083
H -3.524561644 5.730165005 0.248617142
H -3.482711554 6.643754482 1.759800196
H -3.701029062 2.326450825 1.841879010
H -3.639126539 3.172904015 0.299089551
C -1.099166751 5.650404453 2.741954327
H -1.301124215 4.425092697 4.527807236
C -1.206496119 3.128099680 2.793997049
H -0.000918786 5.628052711 2.760299444
H -1.407943606 6.577943325 3.243077040
H -1.581953406 2.261183500 3.350149870
H -0.110209875 3.064532518 2.805580378
H -1.194110990 6.493449688 0.740970016
C -1.151182532 4.335425377 0.585503399
H -1.490068078 4.329725742 -0.455763549
H -0.056507893 4.286787033 0.556630313

=============================

dapd-T2

==============================

Ir	-0.452900767	0.391710579	-1.421792746
N	-2.668643713	0.644884169	-2.224530220
C	-2.750653028	1.882698774	-2.727835417
C	-3.754170895	0.030753724	-1.692969322
C	-3.975982904	2.633809566	-2.621497631
C	-4.997706890	0.604959369	-1.692722440
C	-5.139864922	1.945768833	-2.142655134
H	-3.575688601	-0.962128878	-1.288748622
H	-5.860738277	0.060742877	-1.319868326
N	1.653716326	0.140540615	-0.357384622
C	2.027745962	0.787119687	0.773992598
C	2.050288200	-1.114825368	-0.598728597
C	2.874568462	0.229044437	1.695023060
C	2.803456545	-1.845065832	0.387373686
C	3.275287628	-1.125249028	1.534777999
H	1.631492615	1.792547584	0.888108492
H	3.209879875	0.798339427	2.557285547
C	0.423327237	-1.091000438	-2.493162155
C	1.618105292	-1.635764837	-1.914858341
C	0.014227741	-1.628208041	-3.747397423
C	2.400244951	-2.569885969	-2.609269142
C	0.789570153	-2.606691599	-4.378729820
C	1.995991349	-3.075894117	-3.844990253
C	-0.258432359	1.808050394	-2.860911131
C	0.941035807	2.302114010	-3.447993994
C	-1.491444230	2.359171629	-3.342567682
C	0.881060481	3.246784687	-4.477487564
C	-1.510814667	3.261771202	-4.416170120
C	-0.329870433	3.721138954	-4.996790886
C	-4.096129894	4.022690296	-2.905092955
H	-3.212552309	4.589433193	-3.168492794
C	-6.380920887	2.628167391	-2.077551365
H	-7.256025314	2.083766460	-1.731328726
C	-5.312529564	4.665765762	-2.810162783
H	-5.377007484	5.728471756	-3.026532412
C	-6.471845150	3.959415436	-2.417998075
H	-2.457590818	3.584271908	-4.835396290
H	1.813313127	3.613002062	-4.904758930
C	-0.352253795	4.685006618	-6.158703327
H	0.305646181	5.542989254	-5.977728844
H	-0.005319800	4.205631733	-7.082641125
H	-1.360758543	5.067370415	-6.342502594
C	2.303005934	1.825997353	-3.003996134
H	2.453796148	1.989250898	-1.933881998
H	2.418950081	0.750036418	-3.170076847
H	3.095637321	2.346283674	-3.550797462
C	2.822265863	-4.105848312	-4.575849056
H	3.841950417	-4.152873993	-4.181817055
H	2.384375334	-5.107758999	-4.477227211
H	2.882057428	-3.886667252	-5.647367477
C	-1.238940716	-1.162174821	-4.447545052
H	-1.394464731	-1.716205955	-5.378624439
H	-2.120949507	-1.290900469	-3.814985037
H	-1.184708476	-0.095125295	-4.686071396
H	0.451636732	-3.002110243	-5.335358620
H	3.358066082	-2.881137371	-2.204695463
C	4.077724457	-1.787819743	2.498428345
H	4.442265511	-1.218959928	3.350247860
C	3.070693016	-3.241461754	0.324849844
H	2.641244173	-3.829712391	-0.475552976
C	3.836466789	-3.863252163	1.289336443
H	4.022123337	-4.931553841	1.220639110
C	4.368420601	-3.128695488	2.373766661
C	-1.812394261	0.517933607	1.794359684
O	-0.914196253	-1.000431657	0.186468974
C	-1.391782045	-0.747811735	1.343407631
C	-1.756145239	1.736896873	1.092643499
O	-1.260577083	1.897151947	-0.072235316
H	-2.234603167	0.555822432	2.789551735
H	-7.426770210	4.474505901	-2.358561039
H	4.980626106	-3.627632380	3.119798899
C	-2.106632233	-3.033266783	5.029767036
C	-0.651903212	-2.876863718	4.537449837
C	-0.203372091	-4.173319817	3.831453800
H	0.006628386	-2.673068285	5.392551422
C	-0.569960356	-1.694656372	3.547592878
C	-3.030780077	-3.312423229	3.824612141
H	-2.429880381	-2.121861935	5.551712513
H	-2.174103260	-3.855748653	5.754852295
C	-2.580838919	-4.610466480	3.123119116
H	-4.066840172	-3.418836832	4.172878265
C	-2.954021931	-2.131383181	2.830621243
C	-1.129491925	-4.443834782	2.626258135
H	-3.246860266	-4.833511353	2.278558731
H	-2.650831223	-5.458776951	3.817953348

H	-0.807958782	-5.360701084	2.114962578
C	-1.050334811	-3.262385845	1.634531140
H	0.837519348	-4.078234196	3.494537830
H	-0.237805694	-5.017416954	4.534280300
H	-0.859462440	-0.766689301	4.054928780
H	0.463387430	-1.558745027	3.203849792
C	-1.495865226	-1.945140481	2.316828012
H	-0.028538264	-3.152858019	1.256736755
H	-1.683397889	-3.456404686	0.760651946
H	-3.308760166	-1.216886640	3.320381165
H	-3.622052193	-2.312602997	1.977704525
C	-2.358031750	2.999181986	1.752527118
C	-4.522550106	4.033891678	2.618804932
C	-3.855668306	4.296638489	3.986199141
C	-2.344329119	4.540357113	3.783606768
H	-4.015106678	3.440229654	4.656047344
H	-4.316164494	5.168686867	4.470270634
C	-4.315142632	5.256533623	1.700672626
H	-5.596644402	3.854302168	2.761038303
C	-3.887003183	2.784836531	1.969742179
C	-2.802604437	5.492804050	1.500019312
H	-4.802782536	5.086566448	0.731524587
H	-4.783181190	6.146342754	2.144308567
H	-4.056331158	1.907395005	2.605772734
H	-4.365869999	2.573967695	1.005006075
C	-2.140647173	5.764452457	2.867259502
H	-1.867507935	4.721699238	4.756155014
C	-1.702624917	3.293029547	3.134889603
H	-1.068421602	5.963824272	2.736078024
H	-2.577652931	6.659465313	3.331224203
H	-1.817027926	2.434854984	3.806884289
H	-0.623566449	3.449928522	3.002960205
H	-2.649812460	6.355988026	0.838941813
C	-2.165268898	4.244467258	0.851032495
H	-2.612365246	4.058897972	-0.130993247
H	-1.095357299	4.410224438	0.676494420

Supplementary Discussion

Benchmark of functionals To ensure our calculation results are not only valid for the given DFT methods, we re-calculated all structures using PBE0, B3LYP and B3LYP-D3. The calculated energy gaps between two triplet states with different functionals are summarized in Supplementary Figure 2. The results showed that our conclusions can be reproduced with any of these methods: In all cases, the (piq)2Ir(dend) exhibits a meaningfully large energy gap compared with other dopants indicating that the direct bulkiness of 3,7-diethylnonane-4,6-dione(dend) ligand is valid.

Conformational Isomers Search on the dend ligand To understand the different dend ligand effects as a function of the conformations, we calculated gauche conformers of 3-pentyl substituents in the dend ligand. Not surprisingly, the calculated 3MLCT structure and 3MC structure of gauche conformers have 4.8 kcal/mol and 2.6 kcal/mol higher energies, respectively, than the anti-conformer. Of course, this leads to the slightly decreased energy gap between the two-state of 14.7 kcal/mol, but it is still higher than what was found with the other ligands. The triplet state of the other gauche conformer is higher in energy than the anti conformer with a similar energy gap of 16.4 kcal/mol. These results suggest that the effect of the dend ligand still exists in its various conformational isomers.

Relaxed coordinate scan of dopants To understand whether the transition state between 3MLCT and 3MC state exist or not, we performed the linear scan calculation by elongating two Ir–N bonds in four dopants. The calculated results clearly show that the transition state between 3MLCT and 3MC state cannot be found through a simple PES scan, although two excited states are clearly optimized having the highest energy gap in the (piq)2Ir(dend) dopant.

Supplementary Methods

General Commercially available chemicals were used without further purification after purchase from Sigma-Aldrich and Tokyo Chemical Industry (TCI). NMR spectra recorded on Bruker ASCEND 500 (500MHz for ^{1}H NMR, 125 MHz for ^{13}C NMR). Chemical shift are given in ppm with the residual solvent signal as internal standard (dichloromethane at 5.32 ppm and 54.00 ppm, respectively). Mass spectrometry (MS) analysis was performed with LCMS-IT-TOF.

Synthesis of (piq)$_2$Ir(acac) The mixture of (piq)$_2$ Ir dimer 1.37g (1.0 mmol), Na_2CO_3 1.05g (9.9 mmol), acac 0.99g (9.9 mmol) and 2-ethoxyethanol (50 mL) was stirred at 80 ºC for 12 hours. After cooling to room temperature, the reaction mixture was diluted with dichloromethane (100 mL). The combined organic phases were washed with brine, dried over anhydrous $MgSO_4$, filtered, and concentrated under reduced pressure. The crude product was purified by silica gel chromatography (CH_2Cl_2 : Hexane = 4:6) and dried under vacuum to afford desired products as red solid (0.45g, 30%). ^{1}H-NMR (500MHz, CD_2Cl_2) : δ (ppm) = 8.99-8.98 (m, 2H), 8.25 (d, 2H, 6.5Hz), 7.98(s, 2H), 7.90-7.88(m, 2H), 7.72-7.70(m, 4H), 7.32(d, 2H, 6Hz), 6.55(s, 2H), 4.93(s, 1H), 2.34(s, 6H), 1.56(s, 6H), 1.39(s, 6H). ^{13}C-NMR (125MHz, CD_2Cl_2): δ (ppm) = 185.20, 169.64, 149.22, 147.35, 144.51, 141.35, 137.55, 131.38, 130.98, 130.70, 128.78, 127.79, 127.56, 126.87, 118.46, 100.27, 28.58, 24.18, 21.35. MS(m/z):$[M+H]^+$ calculated for $C_{39}H_{36}IrN_2O_2$, 757.240; found, 759.240.

Synthesis of (piq)$_2$Ir(tmhd) This complex was synthesized by the same method for the (piq)$_2$Ir(acac), except that tmhd was used instead of acac. ^{1}H-NMR (500MHz, CD_2Cl_2) : δ (ppm) = 8.97-8.95 (m, 2H), 8.13 (d, 2H, 6.5Hz), 7.96(s, 2H), 7.86-7.83(m, 2H), 7.67-7.66(m, 4H), 7.24 (d, 2H, 6Hz), 6.57(s, 2H), 5.18(s, 1H), 2.35(s, 6H), 1.42(s, 6H), 0.62(s, 18H). ^{13}C-NMR (125MHz, CD_2Cl_2): δ (ppm) = 196.64, 169.73, 149.27, 147.29, 145.93, 141.37, 137.39, 131.00, 130.74, 130.30, 128.65, 127.60, 127.45, 127.36, 126.96, 118.08, 89.75, 41.41, 28.16, 24.27, 21.41. MS(m/z):$[M+H]^+$ calculated for $C_{45}H_{48}IrN_2O_2$, 841.334; found, 841.334.

Synthesis of (piq)$_2$Ir(dapd) This complex was synthesized by the same method for the (piq)$_2$Ir(acac), except that dapd was used instead of acac. ^{1}H-NMR (500MHz, CD_2Cl_2) : δ (ppm) = 8.98-8.96 (m, 2H), 8.08 (d, 2H, 6.5Hz), 7.95(s, 2H), 7.85-7.83(m, 2H), 7.69-7.65(m, 4H), 7.23 (d, 2H, 6.5Hz), 6.57(s, 2H), 5.02(s, 1H), 2.36(s, 6H), 1.64(s, 6H), 1.49(d, 6H, 12Hz), 1.42(s, 6H), 1.36(d, 6H, 11.5Hz), 1.20(s, 12H). ^{13}C-NMR (125MHz, CD_2Cl_2) : δ (ppm) = 194.00,

169.76, 149.30, 147.22, 146.14, 141.50, 137.40, 130.85, 130.67, 130.25, 128.72, 127.52, 127.48, 127.29, 127.05, 118.01, 89.24, 43.46, 40.06, 37.12, 29.03, 24.24, 21.39. MS(m/z):[M+H]$^+$ calculated for $C_{57}H_{60}IrN_2O_2$, 997.428; found, 997.428.

Synthesis of (piq)$_2$Ir(dend) This complex was synthesized by the same method for the (piq)$_2$Ir(acac), except that dend was used instead of acac. ^{1}H-NMR (500MHz, CD_2Cl_2) : δ (ppm) = 8.96-8.94 (m, 2H), 8.21 (d, 2H, 6.5Hz), 7.98(s, 2H), 7.84-7.82(m, 2H), 7.69-7.67(m, 4H), 7.24(d, 2H, 6.5Hz), 6.57(s, 2H), 4.89(s, 1H), 2.35(s, 6H), 1.61-1.55 (m, 2H), 1.41 (s, 6H), 1.30-1.24(m, 2H), 1.18-1.11(m, 2H), 0.94-0.87(m, 4H), 0.40(t, 6H, 7.5Hz),-0.11(t, 6H, 7.5H). ^{13}C-NMR (125MHz, CD_2Cl_2): δ (ppm) = 190.60, 169.68, 149.33, 147.47, 145.57, 141.59, 137.84, 131.10, 130.79, 130.59, 128.83, 127.66, 127.45, 127.35, 127.10, 118.01, 100.96, 55.70, 27.23, 26.82, 24.27, 21.40, 12.01, 11.62. MS(m/z):[M+H]$^+$ calculated for $C_{47}H_{52}IrN_2O_2$, 869.365; found, 869.365.
